# Characterization of Modification Patterns, Biological Function, Clinical Implication, and Immune Microenvironment Association of m^6^A Regulators in Pancreatic Cancer

**DOI:** 10.3389/fgene.2021.702072

**Published:** 2021-09-17

**Authors:** Kun Fang, Hairong Qu, Jiapei Wang, Desheng Tang, Changsheng Yan, Jiamin Ma, Lei Gao

**Affiliations:** ^1^ Department of Surgery, Yinchuan Maternal and Child Health Hospital, Yinchuan, China; ^2^ Department of Gynaecology, Yinchuan Maternal and Child Health Hospital, Yinchuan, China; ^3^ Department of Pathology, Yinchuan Maternal and Child Health Hospital, Yinchuan, China; ^4^ Central Laboratory, Department of Gastroenterology, First Affiliated Hospital of Harbin Medical University, Harbin, China; ^5^ Central Laboratory, Department of Pancreatic and Biliary Surgery, The First Affiliated Hospital of Harbin Medical University, Harbin, China

**Keywords:** pancreatic cancer, N6-methyladenosine regulators, prognosis, immune microenvironment, immunotherapy

## Abstract

**Objective:** N^6^-methyladenosine (m^6^A) modification may modulate various biological processes. Nonetheless, clinical implications of m^6^A modification in pancreatic cancer are undefined. Herein, this study comprehensively characterized the m^6^A modification patterns in pancreatic cancer based on m^6^A regulators.

**Methods:** Genetic mutation and expression pattern of 21 m^6^A regulators and their correlations were assessed in pancreatic cancer from TCGA dataset. m^6^A modification patterns were clustered using unsupervised clustering analysis in TCGA and ICGC datasets. Differences in survival, biological functions and immune cell infiltrations were assessed between modification patterns. A m^6^A scoring system was developed by principal component analysis. Genetic mutations and TIDE scores were compared between high and low m^6^A score groups.

**Results:** ZC3H13 (11%), RBM15B (9%), YTHDF1 (8%), and YTHDC1 (6%) frequently occurred mutations among m^6^A regulators. Also, most of regulators were distinctly dysregulated in pancreatic cancer. There were tight crosslinks between regulators. Two m^6^A modification patterns were constructed, with distinct prognoses, immune cell infiltration and biological functions. Furthermore, we quantified m^6^A score in each sample. High m^6^A scores indicated undesirable clinical outcomes. There were more frequent mutations in high m^6^A score samples. Lower TIDE score was found in high m^6^A score group, with AUC = 0.61, indicating that m^6^A scores might be used for predicting the response to immunotherapy.

**Conclusion:** Collectively, these data demonstrated that m^6^A modification participates pancreatic cancer progress and ornaments immune microenvironment, providing an insight into pancreatic cancer pathogenesis and facilitating precision medicine development.

## Introduction

Pancreatic cancer represents the most lethal malignancy globally, characterized by high intra-tumoral heterogeneity and undesirable survival outcomes (Jain and Dudeja, 2021). Despite the improvement in standard of care, survival outcomes are extremely undesirable with a 5 year survival rate <10% and median survivals <1 year (Qin et al., 2020). The existing therapies provide only limited efficacy. Despite surgical resection as the main therapeutic strategy for pancreatic cancer, merely 10–15% of newly diagnosed patients are qualified (Peng et al., 2019). Over 50% of subjects are diagnosed at locally advanced or metastatic stages (O'Reilly et al., 2020). Specially, traditional chemotherapy for advanced or metastatic patients merely provides months of overall survival (OS) benefit (Ho et al., 2020). Due to the undesirable clinical outcomes, novel treatment strategies are urgently required. Pancreatic cancer with similar morphology usually displays distinct clinical characteristics, response to therapies and survival outcomes (Bailey et al., 2016). Currently, molecular subtypes have been proposed for guidance of preclinical and clinical management, prediction of first-rank treatment strategies and minimum of treatment-relevant death risk and cost in pancreatic cancer (Collisson et al., 2019). Nevertheless, so far, molecular subtyping does not inform therapeutic decisions.

N^6^-methyladenosine (m^6^A), a dynamic and reversible process, represents the most plentiful posttranscriptional methylation modification of mRNAs in eukaryotic species (Zhang et al., 2020). It occurs in the RRACH sequence (where *R* = A or G, *H* = A, C, or U). m^6^A methylation modulates nearly each step of RNA metabolism like RNA splicing, stability, decay, and translation. Aberrant m^6^A levels alters target gene expression and cellular processes and physiological functions, thereby affecting cancer progression (He et al., 2019). This modification is mainly controlled by three kinds of regulators: methyltransferases (“writers”), demethylases (“erasers”) as well as binding proteins (“readers”). Accumulating evidence has reported the carcinogenesis of m^6^A regulators in pancreatic cancer. For instance, upregulating m^6^A writer METTL14 may promote growth and metastases of pancreatic cancer by mediating PERP mRNA m^6^A (Wang et al., 2020). Nevertheless, it remains limited understanding on the global landscape and dynamic changes of m^6^A regulators in pancreatic cancer. Immune microenvironment exerts an important role in tumor progress and treatment effects for pancreatic cancer (Hegde et al., 2020). Comprehending immune microenvironment and its regulators assist enhance immunotherapy (Torphy et al., 2020). For example, targeting m^6^A eraser ALKBH5 enhances the responsiveness to anti-PD-1 therapy through modulating tumor immune microenvironment (Li et al., 2020). Associations between m^6^A regulators and immune microenvironment have been preliminarily characterized in pancreatic cancer (Xu et al., 2021). Nonetheless, m^6^A regulators-mediated methylation modification patterns and immune microenvironment are ambiguous in pancreatic cancer.

Here, this study systematically assessed m^6^A modification patterns in pancreatic cancer according to m^6^A regulatory genes and their correlations to immune microenvironment. Also, we developed a m^6^A scoring system for quantifying the m^6^A modification patterns in each specimen. These findings might enhance the comprehension on immune microenvironment characteristics as well as make more effective immunotherapeutic strategy.

## Materials and Methods

### Data Acquisition and Preprocessing

RNA sequencing profiling and copy number variation of pancreatic cancer were retrieved from The Cancer Genome Atlas (TCGA) via the UCSC Xena (https://gdc.xenahubs.net/). Meanwhile, the matched clinical data were acquired via cgdsr package. Genomic mutation data of pancreatic cancer containing somatic mutation were also obtained from TCGA database via TCGAbiolinks package (Colaprico et al., 2016). Use Mutation landscape of patients was characterized by maftools package. Also, expression profiles of two pancreatic cancer cohorts (PACA-AU and PACA-AU) were downloaded from ICGC cohort (https://dcc.icgc.org/projects). Specific clinical information was listed in Table 1. To maintain data consistency, sva package was applied for performing batch correction on the pancreatic cancer transcriptome data from TCGA and ICGC databases (Leek et al., 2012). The GSE79668 dataset containing RNA-seq and clinical information of 51 pancreatic cancer patients was downloaded from the Gene Expression Omnibus (GEO) repository (https://www.ncbi.nlm.nih.gov/gds/) (Kirby et al., 2016).

**TABLE 1:** Specific clinical information of pancreatic cancer patients.

Characteristics	TCGA	ICGC: PACA-AU	ICGC: PACA-AU
Sex			
Female	80	88	43
Male	97	109	47
NA	0	37	1
Age			
≥60	123	115	67
<60	54	53	22
Status			
Dead	92	152	58
Alive	85	45	32
NA	0	37	1

### Unsupervised Clustering Analysis

Expression profiles of 21 m^6^A regulators were extracted from TCGA and ICGC datasets as well as GSE79668 dataset. RCircos package was utilized for plotting the chromosome distribution of these regulators in chromosomes. Distinct m^6^A modification patterns were clustered according to expression of m^6^A regulators using unsupervised clustering analysis by ConsensusClusterPlus package (Wilkerson and Hayes, 2010). Patients were classified for distinct molecular subtypes for further analysis. The distance used for clustering was the Euclidean distance. This analysis was repeated 1,000 times to ensure the stability of clustering. Principal component analysis was applied for validating the accuracy of this classification.

### Gene Set Variation Analysis

GSVA, a non-parametric, unsupervised method, is primarily utilized for estimating activity changes in pathway or biological process in a sample (Hänzelmann et al., 2013). For studying the differences in biological processes of distinct m^6^A modification patterns, GSVA package was applied to perform GSVA enrichment analysis based on gene expression profiles. The “c2.cp.kegg.v6.2” gene set from the Molecular Signatures Database (MSigDB) database (https://www.gsea-msigdb.org/gsea/index.jsp) was set as the reference set (Liberzon et al., 2015).

### Single Sample Gene Set Enrichment Analysis

The infiltration levels of 24 immune cells were estimated in each sample by ssGSEA package. Then, the differences between m^6^A modification patterns were compared with Wilcox test. Univariate cox regression analysis was separately presented for assessing the associations between immune cells and prognosis of pancreatic cancer in each cluster.

### Development of m^6^A Score System

Differentially expressed genes (DEGs) were screened between m^6^A modification patterns from TCGA and ICGC databases by limma package (Ritchie et al., 2015). The thresholds were set as adjusted *p* value < 0.05 and log2 |fold-change| > 0.5. The random forest method was utilized for removing redundant genes based on DEGs using randomForest, ROCR and Hmisc packages. The “meandecreaseaccuracy” parameter was set as the standard selection. Then, survival analysis on the remaining genes was performed. Genes with *p* < 0.05 were significantly related to survival outcomes of pancreatic cancer. By cox regression model, genes were separated into two categories according to positive or negative coefficients. m^6^A score was determined using the following formula: m^6^A score = scale(∑X−∑Y). X represented the expression value of the gene set for which regression coefficient was positive. Meanwhile, Y represented the expression value of the gene set for which regression coefficient was negative. Based on the median of m^6^A score, pancreatic cancer specimens were stratified into high and low m^6^A score groups. Kaplan-Meier curves and log-rank tests were performed for assessing the overall survival (OS) differences between groups.

### Association Between m^6^A Score and Biological Pathways

Pearson analysis was performed for assessing associations between m^6^A score and several key biological pathways including immune checkpoints, antigen processing and presentation, EMT1, EMT2, EMT3, and other epithelial-mesenchymal transition (EMT) markers, DNA damage repair, mismatch repair, nucleotide excision repair, and the like.

### Copy Number Variation Analysis

The GISTIC method was employed for detecting the shared copy number change area in all samples based on the SNP6 CopyNumber segment data. The parameters were set as: Q ≤ 0.05 was the change significance standard and the confidence level was 0.95 when determining the peak interval. The analysis was presented through MutSigCV function of GenePattern (https://cloud.genepattern.org/gp/pages/index.jsf) online tool.

### Assessment of T Cell Dysfunction and Exclusion

TIDE (http://tide.dfci.harvard.edu) was employed for assessing the response to immune checkpoint blockade (ICB) (Jiang et al., 2018). TIDE score of each specimen was determined. Receiver operating characteristic curve (ROC) was then carried out for evaluating the efficacy of m^6^A scores for predicting the response to immunotherapy, and the area under the curve (AUC) was quantified with pROC package.

### Statistical Analysis

Statistical analysis was achieved with R language (version 3.6.1) and appropriate packages. Wilcox test was applied for comparing the differences between groups. *p* < 0.05 indicated statistically significance.

## Results

### Landscape of Genetic Mutation and Expression of m^6^A Regulators in Pancreatic Cancer

**FIGURE 1:**
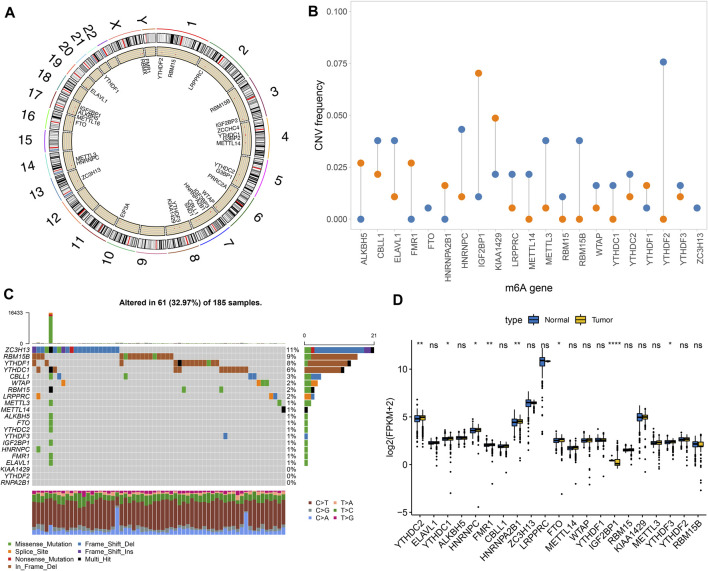
Landscape of genetic mutation and expression patterns of m^6^A regulators in pancreatic cancer. **(A)** The locations of m^6^A regulators on the chromosomes. **(B)** The distribution of CNV frequency of m^6^A regulators. Blue indicates deletion and orange indicates amplification. The height of bar indicates the variation frequency. **(C)** Landscape of somatic mutation of m^6^A regulators. **(D)** Box plot of expression patterns of m^6^A regulators in normal and pancreatic cancer. Ns: not significant; *p < 0.05; **p < 0.01; ****p < 0.0001.

Totally, 21 m^6^A regulators were analyzed in our study. Figure 1A showed the locations of these regulators on the chromosomes. Also, we summarized frequencies of CNV and somatic mutation. In Figure 1B, CNV was common in all regulators. Among them, ALKBH5, FMR1, HNRNPA2B1, IGF2BP1 and KIAA1429 had high frequencies of gain, while other regulators occurred high frequencies of loss. Among 185 pancreatic cancer specimens in TCGA dataset, 61 occurred somatic mutations (Figure 1C). Among them, ZC3H13 (11%), RBM15B (9%), YTHDF1 (8%), and YTHDC1 (6%) displayed higher genetic mutation frequencies. Also, we compared the expression patterns of these regulators in pancreatic cancer and normal tissues. In Figure 1D, YTHDC2, YTHDC1, HNRNPC, FMR1, FTO, IGF2BP1, and YTHDF3 were significantly dysregulated in pancreatic cancer.

### Characterization of Two m^6^A Methylation Modification Patterns in Pancreatic Cancer

**FIGURE 2:**
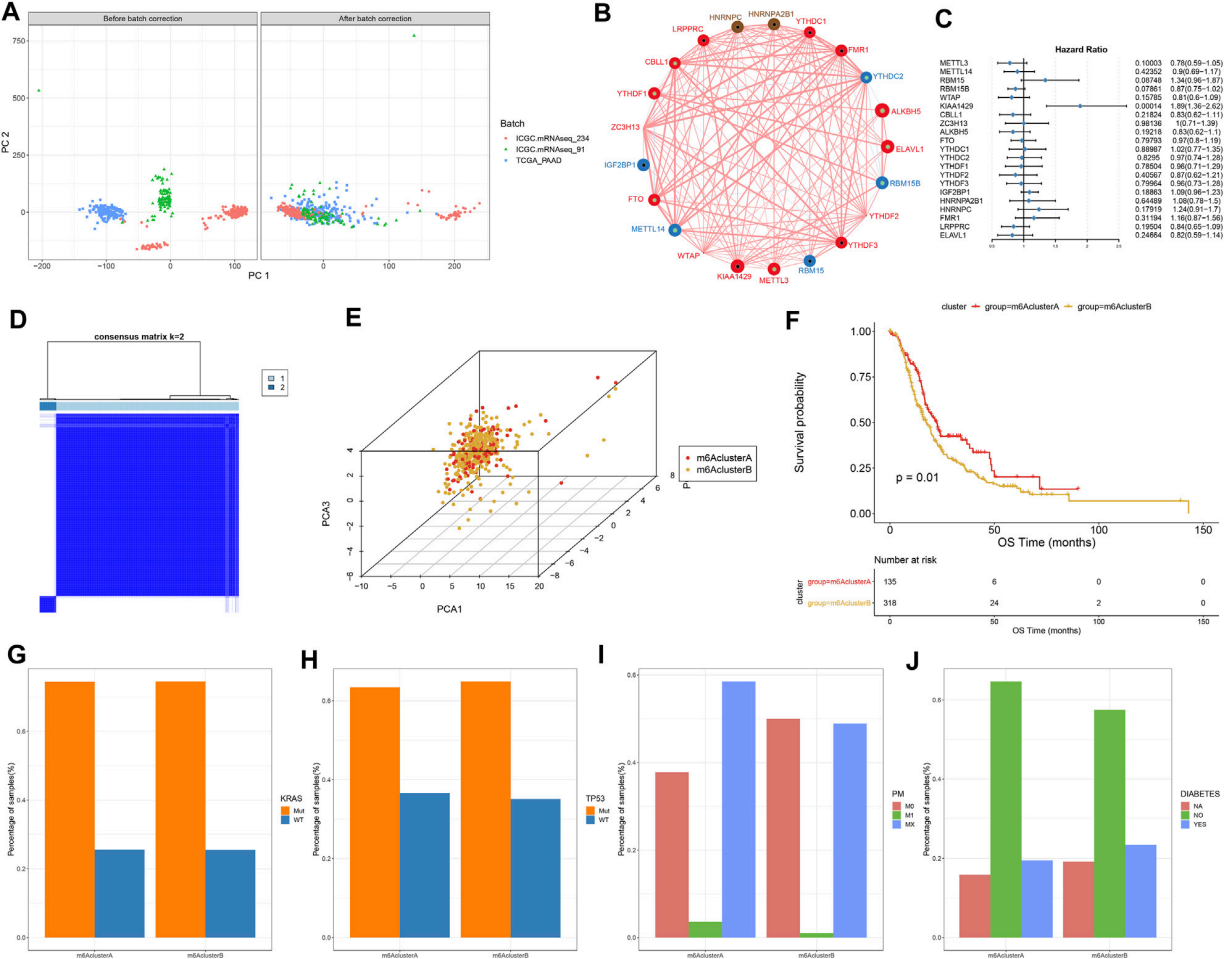
Construction of two m^6^A methylation modification patterns in pancreatic cancer from TCGA and ICGC datasets. **(A)** PCA plots showing before and after batch correction of TCGA and ICGC datasets. **(B)** An interaction network of m^6^A regulators. The size of the circle indicates the impact of each regulator on survival, and the larger the circle, the more relevant its expression is to the prognosis. The green dot in the circle indicates that the regulator is a protective factor for prognosis, and the black dot in the circle indicates that the regulator is a risk factor for prognosis. The lines connecting regulators indicate their interactions, negative correlations are marked in blue, and positive correlations are marked in red. **(C)** Multivariate cox regression analyses for assessing associations of m^6^A regulators with pancreatic cancer prognoses. **(D)** Consensus matrix heatmap when *k* = 2. **(E)** PCA plots showing the differences between m^6^A cluster A and B based on the expression profiling of m^6^A regulators. **(F)** Kaplan-Meier curves of two m^6^A clusters. P was determined with log-rank test. Distributions of **(G)** KRAS mutation, **(H)** TP53 mutation, **(I)** metastasis, and **(J)** diabetes in two m^6^A clusters.

This study integrated RNA-seq data from TCGA and ICGC datasets and batch effects were removed by sva package (Figure 2A). By univariate cox regression analyses, associations between m^6^A regulators and prognoses of pancreatic cancer were evaluated. As a result, ELAVL1, ALKBH5, and KIAA1429 were distinctly correlated to the patients’ prognoses (Table 2). Figure 2B depicted the crosslinks between writers, erasers, and readers, indicating that the interactions between m^6^A regulators might exert a critical role in forming distinct m^6^A modification patterns. Multivariate cox regression analyses revealed that KIAA1429 served as an independent risk factor of pancreatic cancer prognosis among m^6^A regulators (Figure 2C). After extracting the expression profiles of 21 regulators in pancreatic cancer specimens from TCGA and ICGC datasets, unsupervised clustering analysis was carried out with ConsensusClusterPlus package. As a result, 2 modification patterns were clustered (m^6^A cluster A and m^6^A cluster B; Figure 2D; Supplementary Table S1). PCA results demonstrated the prominent differences between clusters based on the expression profiles of m^6^A regulators (Figure 2E). In Figure 2F, samples in m^6^A cluster B displayed poorer OS duration in comparison to those in m^6^A cluster A (*p* = 0.01). However, no significant differences in KRAS mutation (Figure 2G), TP53 (Figure 2H), metastasis (Figure 2I) and diabetes (Figure 2J) were found between clusters. The m^6^A clustering results and survival differences were confirmed in the GSE79668 dataset (Supplementary Figures S1A,B).

**TABLE 2:** Associations between m^6^A regulators and prognoses of pancreatic cancer.

Regulators	Hazard ratio	Lower 0.95% CI	Upper 0.95% CI	*p*
YTHDC2	0.945573	0.804418	1.111497	0.501143
METTL14	0.87198	0.759141	1.001591	0.057076
IGF2BP1	1.102124	0.987532	1.230014	0.095858
RBM15	1.165283	0.893101	1.520415	0.256693
RBM15B	0.895478	0.794545	1.009233	0.06895
ELAVL1	0.745107	0.585853	0.947651	0.020395
YTHDC1	1.034719	0.880397	1.21609	0.677174
ALKBH5	0.755901	0.631814	0.904358	0.002477
FMR1	1.111698	0.934534	1.322447	0.222155
CBLL1	0.943543	0.786501	1.131941	0.534774
ZC3H13	0.992526	0.822421	1.197816	0.937711
LRPPRC	1.066698	0.898443	1.266463	0.456897
FTO	0.938485	0.838581	1.050291	0.276089
WTAP	0.984003	0.784578	1.234117	0.889144
YTHDF1	0.882007	0.721275	1.078557	0.229438
KIAA1429	1.326562	1.066612	1.649864	0.010711
METTL3	0.820477	0.65659	1.025272	0.083879
YTHDF3	1.087143	0.909575	1.299375	0.351575
YTHDF2	0.988164	0.777414	1.256047	0.922555
HNRNPC	1.147295	0.943431	1.395212	0.155486
HNRNPA2B1	1.129765	0.946279	1.348829	0.166181

### Two m^6^A Methylation Modification Patterns Characterized by Distinct Immune Cell Infiltration, Biological Functions, and Genetic Mutations

**FIGURE 3:**
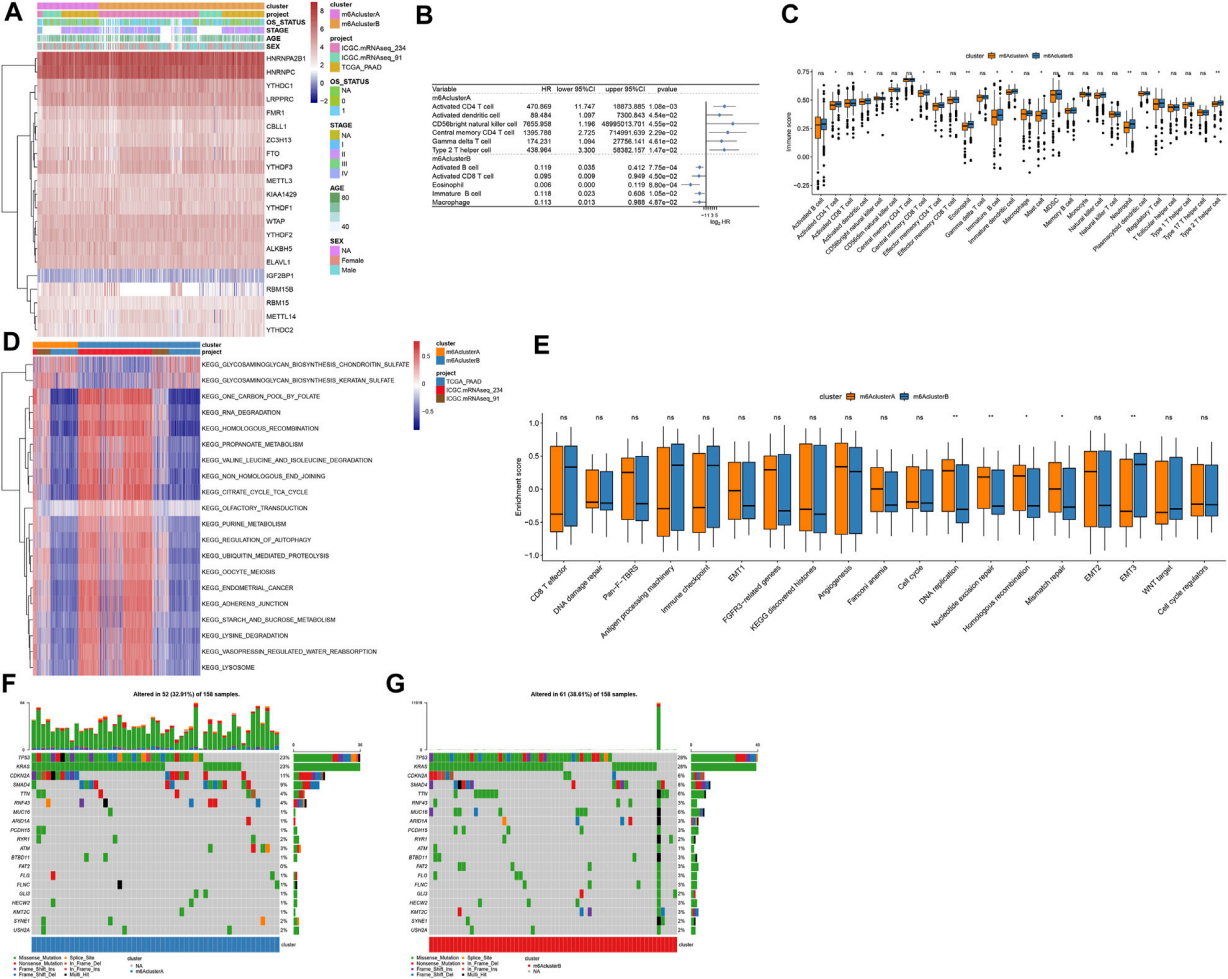
Two m^6^A methylation modification patterns with distinct immune cell infiltration, biological functions, and genetic mutation. **(A)** Heatmap of expression patterns of m^6^A regulators in m^6^A clusters (cluster A and B), OS status, stage, age, and sex. **(B)** Associations between immune cells and prognoses of pancreatic cancer. HR: hazard ratio and CI: confidence interval. **(C)** Box plot of the infiltration levels of immune cells in two m^6^A clusters. Ns: not significant; *p < 0.05; **p < 0.01. **(D)** GSVA enrichment analysis for the activation status of biological pathways in two m^6^A clusters. **(E)** Box plots of the enrichment scores of key biological processes in two m^6^A clusters. **(F)** The somatic mutation landscape of samples in m^6^A cluster A. **(G)** The somatic mutation landscape of samples in m^6^A cluster B.

Figure 3A depicted the expression patterns of 21 m^6^A regulators in two m^6^A methylation modification patterns. By ssGSEA algorithm, we estimated the infiltration levels of 24 immune cells in pancreatic cancer. Univariate cox regression analyses identified that activated CD4 T cell, activated dendritic cell, CD56bright natural killer cell, central memory CD4 T cell, gamma delta T cell and type 2 T helper cell were risk factors of pancreatic cancer prognoses in m^6^A cluster A (Figure 3B). In contrast, we observed that activated B cell, activated CD8 T cell, eosinophil, immature B cell, and macrophage were protective factors of pancreatic cancer prognoses in m^6^A cluster B (Figure 3B). In Figure 3C, m^6^A cluster B was charactered by higher infiltration levels of activated CD4 T cells, activated dendritic cells, central memory CD8 T cells, Effector memory CD4 T cells, eosinophils, immature B cells, immature dendritic cells, mast cells, neutrophils, regulatory T cells and type 2 T helper cells, indicating that there was higher immunogenicity in m^6^A cluster B. T explore the biological behaviors between these different m^6^A modification patterns, GSVA enrichment analysis was carried out. As a result, there were distinct differences in activation of glycosaminoglycan biosynthesis chondroitin sulfate, glycosaminoglycan biosynthesis keratan sulfate, one carbon pool by folate, RNA degradation, homologous recombination, propanoate metabolism, valine leucine and isoleucine degradation, non-homologous end joining, citrate cycle TCA cycle, olfactory transduction, purine metabolism, regulation of autophagy, ubiquitin mediated proteolysis, oocyte meiosis, endometrial cancer, adherens junction, starch and sucrose metabolism, lysine degradation, vasopressin regulated water reabsorption and lysosome between m^6^A clusters (Figure 3D). Furthermore, we found that DNA replication, nucleotide excision repair, homologous recombination and mismatch repair were significantly activated in m^6^A cluster A than cluster B (Figure 3E). However, EMT3 was distinctly activated in cluster B. We also compared the differences in genetic mutations between m^6^A clusters (Figures 3F,G). Higher frequency of mutation was found in cluster B (38.61%) than cluster A (32.91%).

### Construction of m^6^A Gene Clusters in Pancreatic Cancer

**FIGURE 4:**
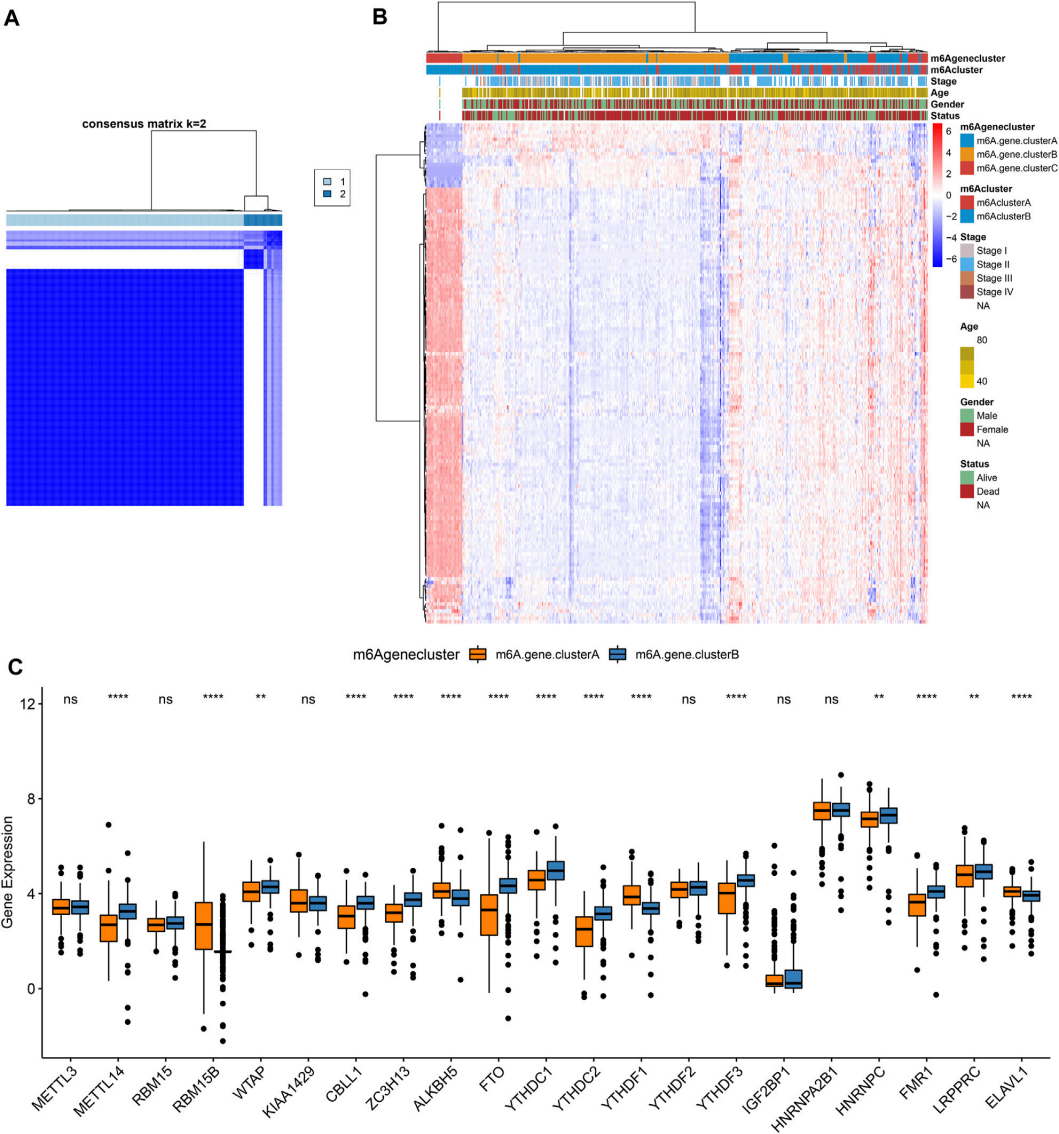
Construction of m^6^A gene clusters in pancreatic cancer. **(A)** Consensus matrix heatmap when *k* = 2. **(B)** Heatmap of two m^6^A gene clusters and expression patterns of m^6^A-related genes. **(C)** Box plot of expression patterns of 21 m^6^A regulators in m^6^A gene cluster A and B. Ns: not significant; **p < 0.01; ****p < 0.0001.

To further study the potential mechanisms of m^6^A clusters, limma package was applied for determining 140 m^6^A-related DEGs with the cutoff values of *p* = 0.05, |log2fold-change| = 0.5 (Supplementary Table S2). By clusterProfiler package, we analyzed KEGG pathways based on the DEGs. Only ribosome was significantly enriched by the DEGs. Furthermore, we performed unsupervised cluster analysis based on the obtained m^6^A-related genes, and stratified the patients into two different m^6^A gene clusters named as m^6^A gene cluster A and B (Figure 4A; Supplementary Table S3). The expression patterns of the m^6^A-related genes were visualized, as shown in Figure 4B. METTL14, WTAP, CBLL1, ZC3H13, FTO, YTHDC1, YTHDC2, YTHDF3, HNRNPC, FMR1, and LRPPRC were distinctly up-regulated in m^6^A gene cluster B while RBM15B, ALKBH5, YTHDF1, and ELAVL1 were significantly up-regulated in m^6^A gene cluster A (Figure 4C).

### Development of a m^6^A Scoring System in Pancreatic Cancer

**FIGURE 5:**
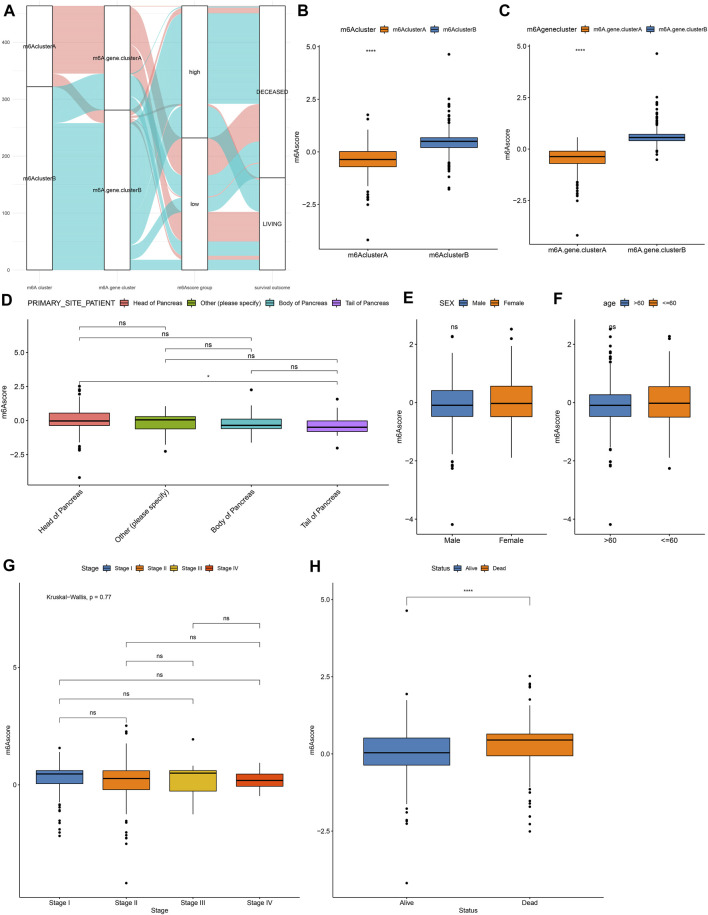
Development of a m^6^A scoring system in pancreatic cancer. **(A)** Alluvial diagram for the relationships of m^6^A modification patterns, m^6^A gene clusters and m^6^A scores. **(B)** Distribution of m^6^A scores in m^6^A cluster A and B. **(C)** Distribution of m^6^A scores in m^6^A gene cluster A and B. Distributions of m^6^A scores in **(D)** different primary sites, **(E)** sex, **(F)** age, **(G)** stage, and **(H)** survival status. Ns: not significant; ****p < 0.0001.

For the m^6^A-related genes, the random forest algorithm was used for eliminating the redundancy of DEGs. The characteristic genes that were most relevant to the classification were screened out, including RABAC1, ALKBH7, DPM3, POLR2I, MBD3, ISOC2, WBSCR16, CUTA, C17orf89, MRPL41, ZNF787, C19orf60, and C19orf43. By cox regression model, we determined the relationships between these genes and prognoses. According to the coefficients, the genes were divided into two categories. With the m^6^A score calculation formula, each pancreatic cancer was scored (Supplementary Table S4). Based on m^6^A score median, we stratified samples into high and low m^6^A score groups (Figure 5A). Higher m^6^A scores were detected in m^6^A cluster B (Figure 5B) and m^6^A gene cluster B (Figure 5C). There were not significant differences in primary sites (Figure 5D), sex (Figure 5E), age (Figure 5F) and stage (Figure 5G) between high and low m^6^A score groups. However, patients with dead status exhibited higher m^6^A score than those with alive status (Figure 5H).

### m^6^A Scores as a Prognostic Factor of Pancreatic Cancer

**FIGURE 6:**
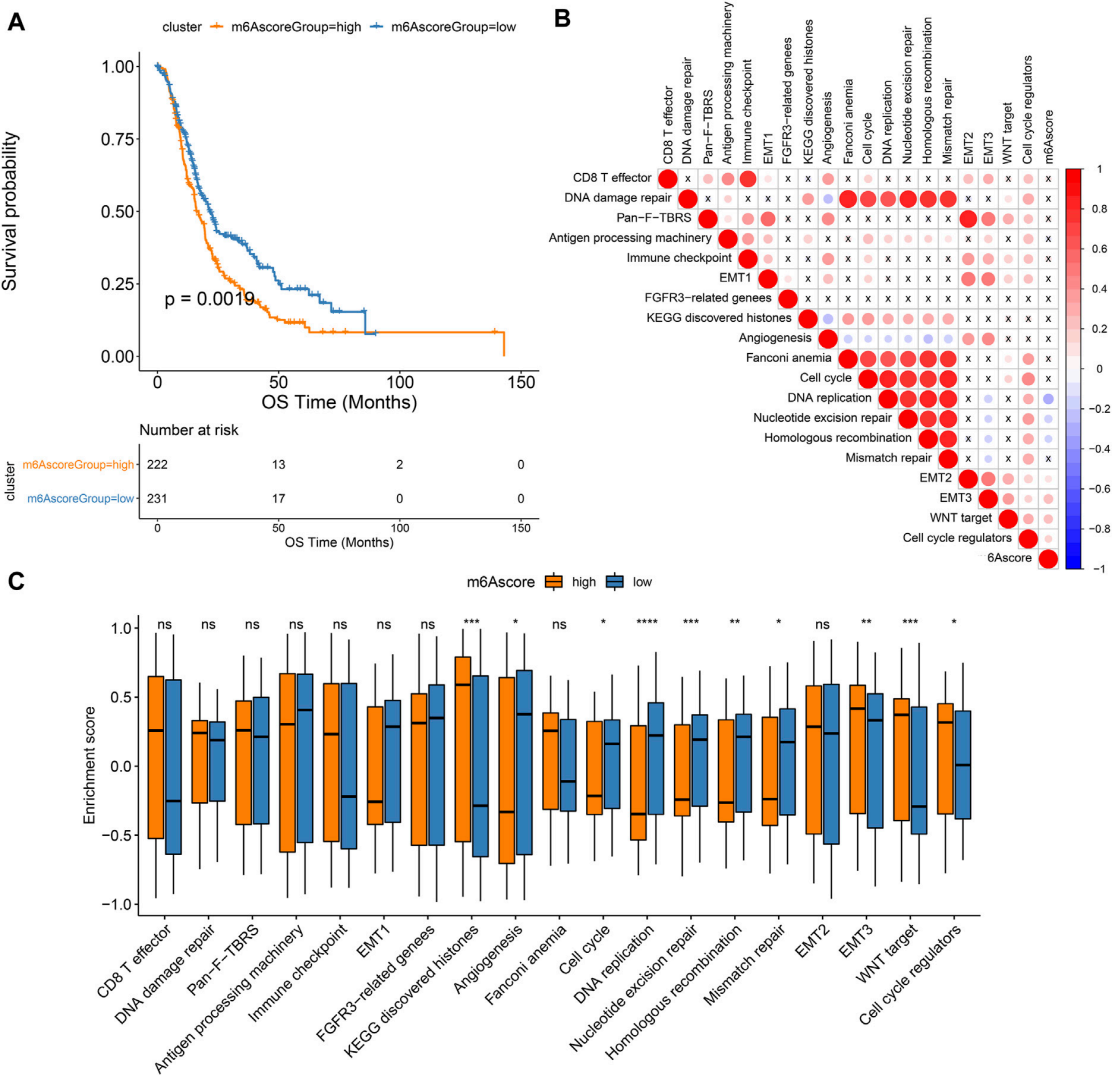
Associations between m^6^A scores and pancreatic cancer prognoses. **(A)** Kaplan-Meier curves of patients with high and low m^6^A scores. **(B)** Correlation between key biological processes and m^6^A scores. **(C)** Box plot of the enrichment scores of key biological processes in high and low m^6^A score groups. Ns: not significant; *p < 0.05; **p < 0.01; ***p < 0.001; ****p < 0.0001.

As shown in Figure 6A, patients in high m^6^A score group displayed a poor prognosis, while those in low m^6^A score group had a good prognosis, indicating that the m^6^A scoring system can provide a good characterization of the prognosis of pancreatic cancer. The prognostic implication of m^6^A score was confirmed in the GSE79668 dataset (Supplementary Figure S1C). In Figure 6B, m^6^A scores were distinctly correlated to DNA replication, nucleotide excision repair, homologous recombination, EMT2, EMT3, WNT target and cell cycle regulators. Furthermore, high m^6^A scores were characterized by activation of histones, EMT3, WNT target and cell cycle regulators, while low m^6^A scores were characterized by angiogenesis, cell cycle, DNA replication, nucleotide excision repair, homologous recombination, and mismatch repair (Figure 6C).

### Assessment of Genetic Mutation Characteristics of High and Low m^6^A Scores

**FIGURE 7:**
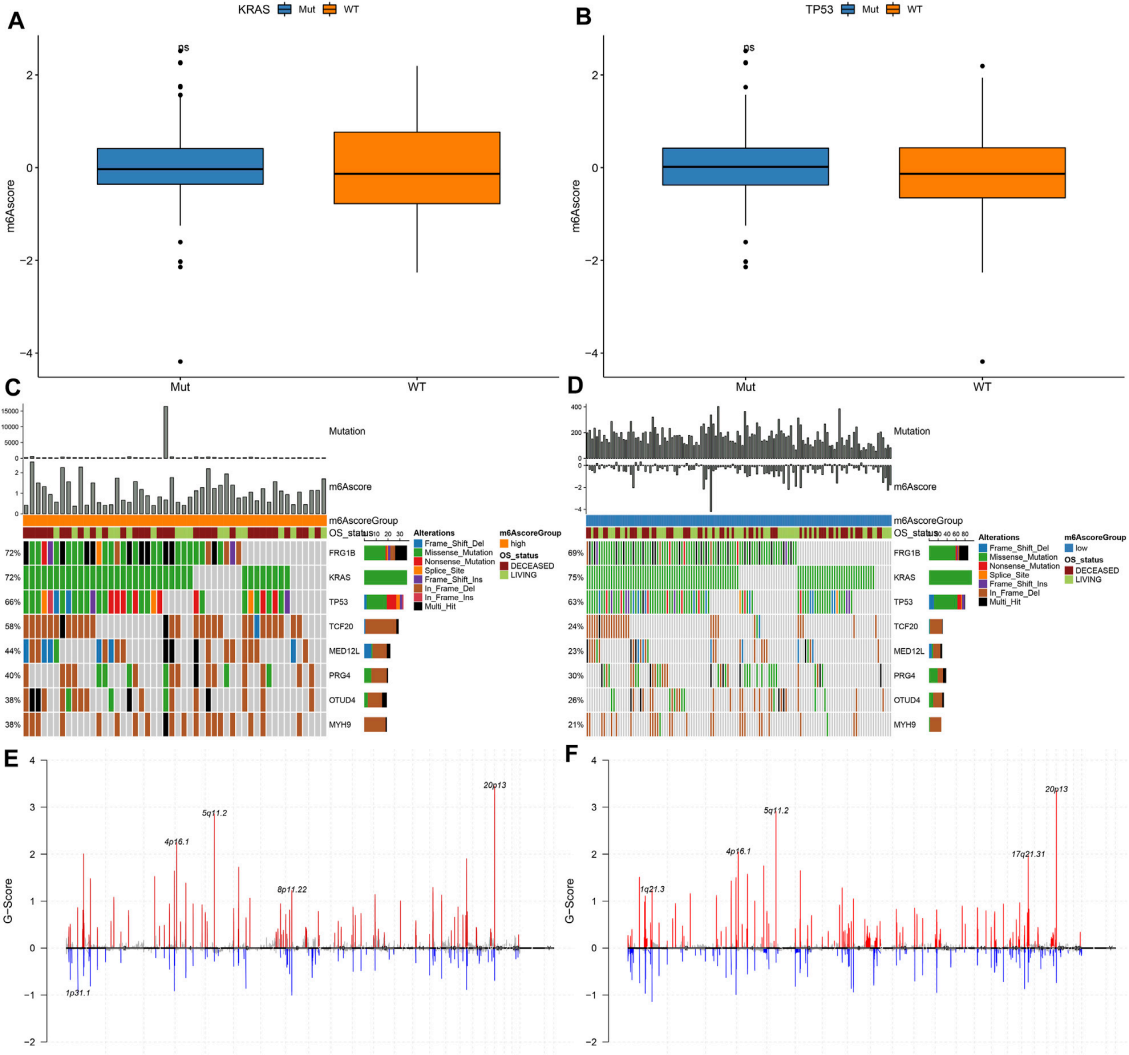
Genetic mutations of samples with high and low m^6^A scores. **(A)** Distribution of KRAS mutation in high and low m^6^A score groups. **(B)** Distribution of TP53 mutation in high and low m^6^A score groups. **(C,D)** The somatic mutation landscape of samples with high and low m^6^A scores. **(E,F)** The CNV landscape of samples with high and low m^6^A scores.

Our analysis found that m6A scores had no significant differences in KRAS mutation (Figure 7A) and TP53 mutation (Figure 7B **)**. We applied maftools package for analyzing the differences in somatic mutations between high and low m^6^A score groups. Figures 7C,D showed the frequencies of genetic mutations in two groups. Both in high and low m^6^A score groups, FRG1B, KRAS, TP53, TCF20, MED12L, PRG4, OTUD4, and MYH9 were the eight most frequently mutated genes. Missense mutation was the main mutation type in pancreatic cancer. Figures 7E,F showed the distributions of CNV regions in two groups.

### m^6^A Score as a Predictive Tool of Immunotherapy Response

**FIGURE 8:**
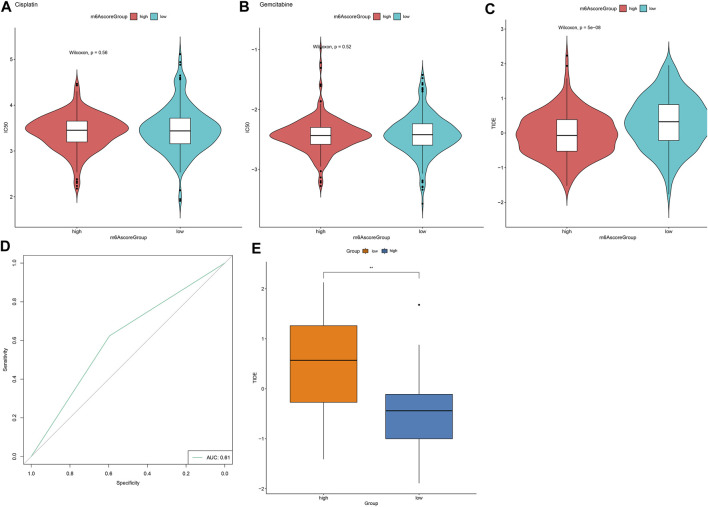
m^6^A score might be used for predicting the response to immunotherapy. **(A,B)** Violin plots for visualizing IC50 values of **(A)** cisplatin and **(B)** gemcitabine in high and low m^6^A score groups. **(C)** Violin plots of TIDE scores in high and low m^6^A score groups. Comparisons between groups were analyzed with Wilcoxon test. **(D)** ROC curves for assessing the response to immunotherapy based on m^6^A scores. **(E)** Validation of TIDE scores in high and low m^6^A score groups in the GSE79668 dataset. **p < 0.01.

We further employed pRRophetic package for estimating IC50 values of chemotherapy drugs (Cisplatin, Gemcitabine) based on the expression profile. There were no significant differences in IC50 values of Cisplatin and Gemcitabine between high and low m6A scores (Figures 8A,B). Furthermore, TIDE scores were determined for evaluating the clinical effects of ICB treatment in high and low m6A score groups based on the mRNA expression profiles. As shown in Figure 8C, TIDE scores of the high m^6^A score group were distinctly lower than low m^6^A score group. AUC reached 0.62, indicating that the m^6^A score might be utilized for predicting the response of immunotherapy (Figure 8D). Difference in TIDE scores between high and low m^6^A score groups was confirmed in the GSE79668 dataset (Figure 8E).

## Discussion

Pancreatic cancer represents a highly lethal malignancy with limited therapeutic options (Liang et al., 2020). Aberrant m^6^A levels participate in modulating cancer malignant phenotypes through affecting the expression of tumor-related genes (Guo et al., 2020). Pancreatic cancer patients with genetic alterations of m^6^A regulators exhibit worse disease-free and OS (Meng et al., 2020). Despite the anti-cancer effects of several m^6^A enzyme inhibitors, more effective m^6^A-related drugs and treatment options required to be further probed. Here, we constructed two m^6^A modification patterns, characterized by different survival outcomes, biological functions, and immune cell infiltration. To individually quantify the m^6^A modification, we developed a m^6^A scoring system. High m^6^A scores indicated undesirable clinical outcomes and predicted high sensitivity to respond to immunotherapy in pancreatic cancer.

32.97% pancreatic cancer samples occurred genetic mutations. ZC3H13 (11%), RBM15B (9%), YTHDF1 (8%), and YTHDC1 (6%) frequently occurred genetic mutations in pancreatic cancer. Frame shift deletion was the most mutation type of ZC3H13 and in-frame deletion was the most mutation classification of RBM15B, YTHDF1, and YTHDC1. Crosslink among writers, erasers. and readers participates in cancer pathogenesis and progress (Ma et al., 2019). Here, tight crosslinks between m^6^A regulators were found in pancreatic cancer. Based on the expression profiles of m^6^A regulators, we constructed two m^6^A clusters with distinct OS duration. Compared with m^6^A cluster A, we observed that m^6^A cluster B was charactered by higher infiltration levels of activated CD4 T cells, activated dendritic cells, central memory CD8 T cells, Effector memory CD4 T cells, eosinophils, immature B cells, immature dendritic cells, mast cells, neutrophils, regulatory T cells, and type 2 T helper cells, demonstrating higher immunogenicity in m^6^A cluster B. Consistently, previous studies have reported the interactions between m^6^A and tumor microenvironment of pancreatic cancer. For instance, both arm-level gain and deletion of ALKBH5 is relation to decreased infiltration of CD8 + T cell in pancreatic adenocarcinoma (Tang et al., 2020b).

This study proposed m^6^A score system for quantifying the m^6^A modification pattern of individual pancreatic cancer by PCA algorithm. Lowered m^6^A scores were detected in m^6^A gene cluster A. Furthermore, we found that m^6^A scores were not correlated to clinical characteristics including primary sites, sex, and age. Nevertheless, high m^6^A scores were in relation to depressed OS duration, demonstrating that m^6^A scores might be utilized for predicting pancreatic cancer prognoses. A previous study developed a six-m^6^A-regulator-signature prognostic model that was markedly associated with OS as well as clinical features (pathologic M, N, clinical stages, and vital status) (Hou et al., 2020). To uncover the molecular mechanism behind m^6^A scores, this study evaluated the enrichment scores of cancer-related pathways between high and low m^6^A score groups. High m^6^A scores were characterized by increased activation of EMT3, Wnt targets, and cell cycle regulators. YTHDF2 orchestrates EMT process in pancreatic cancer (Chen et al., 2017). ALKBH5 suppresses pancreatic cancer tumorigenesis through mediation of Wnt pathway (Tang et al., 2020a). Meanwhile, low m^6^A scores were distinctly related to angiogenesis, cell cycle, DNA replication, nucleotide excision repair, homologous recombination, and mismatch repair. Here, both in high and low m^6^A score groups, FRG1B, KRAS, TP53, TCF20, MED12L, PRG4, OTUD4, and MYH9 were the eight most frequently mutated genes. Missense mutation was the main type of mutation in pancreatic cancer. Genomic and transcriptomic research has uncovered key genetic mutations may drive pancreatic cancer initiation and progress, like KRAS driver mutation (beyond 90%) as well as frequently inactivated TP53 tumor suppressor (beyond 50%) (Peng et al., 2019). A previous study constructed a LASSO prognostic model based on the m^6^A regulators and showed that, KRAS mutation status prominently differed between high- and low-risk subgroups in pancreatic cancer (Geng et al., 2020). In our study, no significant differences in KRAS and TP53 mutations were found in high and low m^6^A score groups. Cisplatin and gemcitabine are standard chemotherapy protocols in pancreatic cancer (Liedtke et al., 2020). Nevertheless, chemo-resistance is the most common phenomenon in pancreatic cancer therapy (Herbst and Zheng, 2019). In previous research, up-regulating m^6^A demethylase ALKBH5 may enhance the sensitivity to gemcitabine in pancreatic cancer (Tang et al., 2020a). Furthermore, pancreatic cancer cells with inhibition of m^6^A writer METTL3 displays higher sensitivity to cisplatin and gemcitabine (Taketo et al., 2018). Above research emphasizes key roles of m^6^A regulators in pancreatic cancer resistance. Nevertheless, no significant differences in sensitivity to cisplatin and gemcitabine were detected between high and low m^6^A score groups.

ICB can produce long-lasting clinical effects. However, limited pancreatic cancer patients benefit from these therapies due to low immunogenicity as well as immunosuppressive tumor microenvironment (Macherla et al., 2018). Combining ICB with other modalities like vaccines, chemoradiotherapy, and target therapies possibly overcomes resistance and enhances immune response in pancreatic cancer. TIDE has been developed for predicting ICB response (Jiang et al., 2018). In previous research the efficacy of anti-PD-L1 therapy can be enhanced by m^6^A-binding protein YTHDF1 inhibition (Han et al., 2019). Also, suppression of m^6^A demethylase FTO may enhance the responsiveness to anti-PD-1 blockade (Yang et al., 2019). Here, high m^6^A score group displayed lower TIDE scores, indicating that these patients were more likely to respond to ICB therapies. AUC = 0.61 indicated that m^6^A scores might be utilized for predicting immunotherapy response.

Taken together, this study offered new insights into prolonging pancreatic cancer patients’ survival duration and enhancing the response to immunotherapy, thereby promoting personalized cancer immunotherapy.

## Conclusion

Collectively, these data characterized two distinct m^6^A methylation modification patterns and their associations with immune microenvironment. By comprehensively evaluating individualized m^6^A modification patterns, we may fully understand immune microenvironment characteristics and develop more effective immunotherapeutic options.

## Data Availability

The original contributions presented in the study are included in the article/Supplementary Material, further inquiries can be directed to the corresponding author.

## Abbreviations

OS, overall survival; m6A, N6-methyladenosine; TCGA, the cancer genome atlas; GSVA, gene set variation analysis; MSigDB, molecular signatures database; ssGSEA, single sample gene set enrichment analysis; DEGs, differentially expressed genes; EMT, epithelial-mesenchymal transition; CNV, copy number variation; TIDE, T cell dysfunction and exclusion; ICB, immune checkpoint blockade; ROC, receiver operating characteristic curve; AUC, area under the curve.
