# Supplementary figures and images for: Characterization of Modification Patterns, Biological Function, Clinical Implication, and Immune Microenvironment Association of m6A Regulators in Pancreatic Cancer
Source: Front Genet. 2021 Sep 17;12:702072. doi: 10.3389/fgene.2021.702072 (PMC8484796; doi:10.3389/fgene.2021.702072)

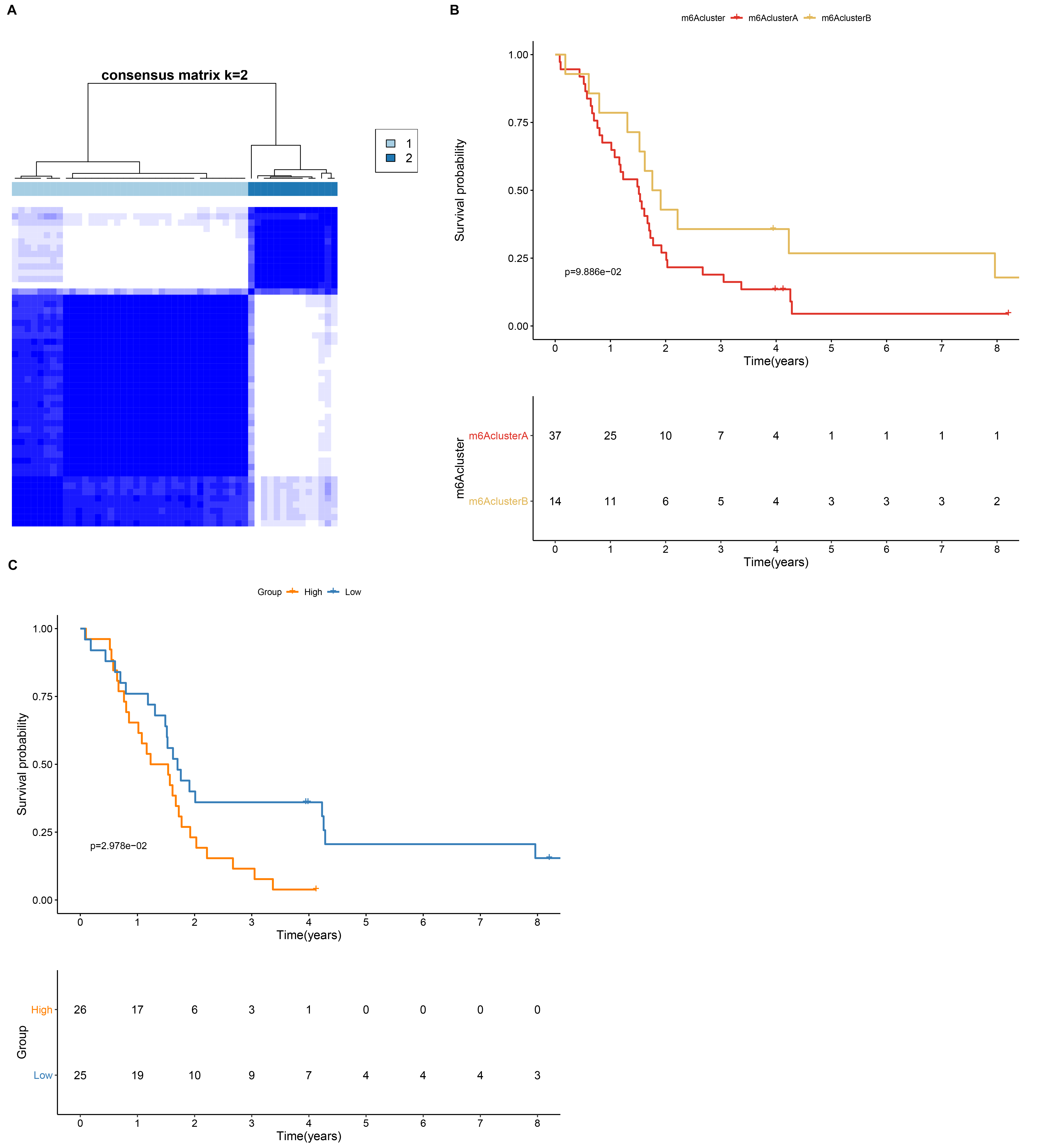

Supplement: Supplementary file 4 [file Image1.TIF]
